# Adherence to secondary prophylaxis and disease recurrence in 536 Brazilian children with rheumatic fever

**DOI:** 10.1186/1546-0096-8-22

**Published:** 2010-07-26

**Authors:** Christina F Pelajo, Jorge M Lopez-Benitez, Juliana M Torres, Sheila KF de Oliveira

**Affiliations:** 1 Pediatric Rheumatology, Floating Hospital for Children at Tufts Medical Center, 800 Washington St, box#190. Boston, MA, 02111, USA; 2 Pediatric Rheumatology, Instituto de Puericultura e Pediatria Martagão Gesteira, Universidade Federal do Rio de Janeiro. Av. Bruno Lobo, 50. Fundão, Rio de Janeiro, 21490-591, Brazil

## Abstract

**Background:**

More than 15 million people worldwide have rheumatic fever (RF) and rheumatic heart disease due to RF. Secondary prophylaxis is a critical cost-effective intervention for preventing morbidity and mortality related to RF. Ensuring adequate adherence to secondary prophylaxis for RF is a challenging task. This study aimed to describe the rates of recurrent episodes of RF, quantify adherence to secondary prophylaxis, and examine the effects of medication adherence to the rates of RF in a cohort of Brazilian children and adolescents with RF.

**Methods:**

This retrospective study took place in the Pediatric Rheumatology outpatient clinic at a tertiary care hospital (Instituto de Puericultura e Pediatria Martagão Gesteira) in Rio de Janeiro, Brazil, and included patients with a diagnosis of RF from 1985 to 2005.

**Results:**

536 patients with RF comprised the study sample. Recurrent episodes of RF occurred in 88 of 536 patients (16.5%). Patients with a recurrent episode of RF were younger (p < 0.0001), more frequently males (p = 0.003), and less adherent (p < 0.0001) to secondary prophylaxis than patients without RF recurrence. Non-adherence to medication at any time during follow-up was detected in 35% of patients. Rates of non-adherence were higher in the group of patients that were lost to follow-up (42%) than in the group of patients still in follow-up (32%) (p = 0.027). Appointment frequency was inadequate in 10% of patients. Higher rates of inadequate appointment frequency were observed among patients who were eventually lost to follow-up (14.5%) than in patients who were successfully followed-up (8%) (p = 0.022). 180 patients (33.5%) were lost to follow up at some point in time.

**Conclusions:**

We recommend implementation of a registry, and a system of active search of missing patients in every service responsible for the follow-up of RF patients. Measures to increase adherence to secondary prophylaxis need to be implemented formally, once non-adherence to secondary prophylaxis is the main cause of RF recurrence. Detection of irregularity in secondary prophylaxis or in appointments should be an alert about the possibility of loss of follow-up and closer observation should be instituted.

## Background

In developing countries, rheumatic fever (RF) is the predominant cause of acquired childhood cardiopathy [1,2]. More than 15 million people worldwide have RF and rheumatic heart disease (RHD) due to RF, with nearly a quarter million deaths occurring annually due to this condition [3-6]. The prevalence of RHD is estimated to be higher in developing than in developed countries, ranging from 24/1,000 to 0.3/1,000, respectively [3,6,7]. It is estimated that 95% of the cases of RHD and deaths related to this disease occur in developing countries [8]. Moreover, significant costs are associated with the treatment of RHD, including heart valve replacement [9].

The severity and prognosis of RHD depends on the extent of cardiac involvement and the frequency of recurrent events [6,10-13]. The risk of RF after an untreated group A beta-hemolytic streptococcal (GABHS) infection in healthy children is around 3% [4,6]; however in children with a previous episode of RF, this risk increases to more than 50%, emphasizing the importance of secondary prophylaxis [14]. Secondary prophylaxis, including the use of benzathine penicillin G, is therefore a critical cost-effective intervention for preventing morbidity and mortality related to RF [3,6,8,9,15,16].

However, ensuring adequate adherence to secondary prophylaxis for RF has been a challenging task, particularly in adolescents; as with most chronic treatments, adherence is usually poor [17-20]. There is no reliable data available with regards to adherence to secondary prophylaxis and the rates of recurrent RF in many developing countries, including Brazil.

The objectives of this large observational study were to describe the rates of recurrent episodes of RF, quantify adherence to secondary prophylaxis, and examine the effects of medication adherence to the rates of RF in a cohort of Brazilian children and adolescents with RF.

## Methods

This retrospective study took place in the Pediatric Rheumatology outpatient clinic at a tertiary care hospital (Instituto de Puericultura e Pediatria Martagão Gesteira) in Rio de Janeiro, Brazil, and included patients with a diagnosis of RF from 1985 to 2005. Information was retrieved from patients' medical records, using a structured data collection sheet.

We identified 548 cases of children and adolescents with a diagnosis of RF. All patients who fulfilled the Jones criteria [12] for the diagnosis of RF were included; 12 patients were excluded from the present analysis because they did not return to the clinic after their first appointment.

Patients were classified as "adherent" to therapy when they did not skip or delay more than one dose of benzathine penicillin G during a 6-month period (interval between appointments). If more than one dose of benzathine penicillin G was delayed or missed during this period, patients were classified as "non-adherent". Administered doses of benzathine penicillin G were recorded on a card given to all patients with RF in Brazil. The classification of adherence contained in the outpatient charts, was primarily based on this card, as well as self-reports when patients had forgotten the card in a specific appointment.

Appointment frequency was considered adequate if the interval between visits was less than 9 months. The interval between appointments in our clinic is normally 6 months for RF patients who are being followed for secondary prophylaxis. If patients had their appointments in intervals longer than 9 months, appointment frequency was considered inadequate. When patients did not return for an appointment for more than 18 months, they were considered lost to follow-up.

We classified a RF episode as recurrent according to standardized World Health Organization criteria [12,21].

The Research Ethics Committee of Instituto de Puericultura e Pediatria Martagão Gesteira approved this project.

### Statistical analyses

The rates of recurrence, non-adherence to secondary prophylaxis, and inadequate frequency to appointments were calculated for the total group, as well as for patients who were successfully followed-up, and for those that were lost to follow-up, separately. Differences in the characteristics of patients who did and did not have a recurrent episode of RF were examined with regards to age, sex, adherence to prophylaxis, frequency of follow-up appointments and follow-up status. Fisher's exact test was used to examine differences between these 2 comparison groups with regards to categorical variables whereas the Student T-test was used to compare differences in various continuous variables. Statistical significance was established with an alpha of 0.05.

## Results

A total of 536 patients with RF comprised the study sample. The average age of the study sample was 13 (± 3.9) years and 53% were girls.

Recurrent episodes of RF occurred in 88 of 536 patients (16.5%). Patients with a recurrent episode of RF were younger, more frequently males, and less adherent to secondary prophylaxis than patients without RF recurrence (table 1). There was a trend to a higher rate of adequate frequency to appointments in the group of patients that did not have a recurrence (p = 0.07). There was not a significant difference between patients who did or did not have a recurrence in relation to loss of follow-up. Within patients who had a recurrence 54.5% were non-adherent to secondary prophylaxis. 31% had a recurrence because prophylaxis was not prescribed, as those patients had not had the diagnosis of RF in a previous episode due to lack of sufficient criteria for the diagnosis. 14.5% of the patients who had recurrences reported adherence to prophylaxis. As expected, non-adherence to secondary prophylaxis had a significant association with recurrences of RF (p < 0.0001).

**Table 1:** Characteristics of patients with and without recurrent episodes of RF

	Patients with recurrent episodes of RF (n = 88)	Patients without recurrent episodes of RF (n = 448)	P value
Age (median, years)	9	14	< 0.0001

Sex (female)	38%	56%	0.003

Non-adherence	54.5%	20%	< 0.0001

Adequate frequency to appointments	79%	90%	0.07

Lost to follow-up	37.5%	29%	0.14

Non-adherence to medication at any time during follow-up was detected in 35% (188 out of 536) of patients. Rates of non-adherence were higher in the group of patients that were lost to follow-up (42%) than in the group of patients still in follow-up (32%) (table 2). The mean age of non-adherent patients was 14.5 years.

**Table 2:** Recurrence, non-adherence, and inadequate frequency to appointments in the total sample and subgroups

	Patients successfully followed-up (n = 356) N (%)	Patients lost to follow-up (n = 180) N (%)	p-value	Total (% in the total group)
Recurrences	55 (15%)	33 (18%)	0.14	88 (16.5%)

Non-adherence	113 (32%)	75 (42%)	0.027	188 (35%)

Inadequate frequency to appointments	28 (8%)	26 (14.5%)	0.022	54 (10%)

Appointment frequency was inadequate in 10% of patients. Higher rates of inadequate appointment frequency were observed among patients who were eventually lost to follow-up (14.5%) than in patients who were successfully followed-up (8%) (table 2).

One hundred eighty patients (33.5%) were lost to follow up at some point in time. The time between the first appointment and the last in this group of patients ranged from 1 month to 13.4 years. The mean time of follow-up in this group was 6.8 years. There were no significant differences in either age or sex in the group lost to follow-up and the group that was successfully followed-up.

## Discussion

Recurrences of RF are directly related to morbidity, mortality and disease progression [22]. 16.5% of patients in our study had a recurrent episode of RF, however recurrence was found in 0.9% of patients in a rural district in north of India [19], and in 0.4% in a study involving 16 developing countries [23]. Rates similar to ours were detected in Chile (17%) in 1993 [24]. In Alexandria (Egypt), in 1998, RF recurrence was found to be 37.3% and the risk factors implied were: living in rural and semi-urban areas, and lack of adherence to secondary prophylaxis [25]. In Australia the implementation of a RF register was associated with a decrease in recurrence rates from 28% (in 1998) to 16% (in 1999) [14]. In contrast to our results, increase in age has been identified as a predictor of RF recurrence in a cross-sectional study in Nepal [2].

In our population, recurrences were associated to non-adherence to secondary prophylaxis in 54.5% of cases. However, in 31% secondary prophylaxis was not prescribed because they did not have a diagnosis of RF on a previous episode, or because prophylaxis was withheld in patients who did not meet Jones criteria. These patients had a previous suspicious episode that did not fulfill Jones criteria, and then presented with a recurrence that made the diagnosis possible, according to the mentioned criteria. After the diagnosis was definitely established, secondary prophylaxis was prescribed. A common and preventable error detected among patients in our study population was precocious use of non-steroidal anti-inflammatory (NSAID) drugs, prescribed before they reached our clinic. This may prevent the natural evolution of polyarthritis, making diagnosis difficult or even impossible, preventing adequate management of patients with secondary prophylaxis and letting them exposed to recurrences [6,25]. This issue has been described in other high-incidence populations [26], and that's why the 2006 New Zealand guidelines for RF consider aseptic monoarthritis as a major criterion when there is a history of prior NSAID use [6].

After each revision of Jones criteria, specificity increased and sensitivity decreased [6]. This occurred because of the decrease of RF incidence in developed countries [3]. Jones criteria should be a guide to help physicians but should not be strictly applied and substitute clinical judgment, as it could result in underdiagnosis of RF in countries where it is still highly incident, as in Brazil [6,13,21,26]. In countries where RF is still endemic or epidemic the risk associated with an eventual recurrence with possible development or aggravation of RHD, surpasses consequences of a false diagnose [3].

Non-adherence to secondary prophylaxis was 35% in the total sample of patients (536). In other studies this rate varies from 10% to 65.7% [5,14,19,23,25,27-32]. Although high, the rate reported in our center corresponds to rates found in other centers. Among patients lost to follow-up, 42% were non-adherent, compared to 32% in the group successfully followed-up (p = 0.027). There is a significant difference within the two groups, and non-adherence to secondary prophylaxis might be used as a precocious sign of the possibility of loss of follow-up.

Factors related to the lack of adherence in other studies were: lower education of the parents, living in rural or semi-urban areas, low parental knowledge about the disease and dissatisfaction of the family with care [25]. As our study was retrospective, we could not analyze the causes of lack of adherence.

To guarantee higher adherence to prophylaxis and appointments, implementation of education and awareness strategies for patients and families may be a solution. According to the KAP (knowledge, attitude, practice) model of promotion of health, knowledge is necessary in order to change individuals' behaviors [17,20]. Other effective strategies for preventing discontinuation of follow-up and of secondary prophylaxis are notification of RF cases, and implementation of mechanisms to identify and localize patients who have been missing appointments [15,30]. Counseling about the relevance of adherence to therapy, how to organize administration of medication, remembering notes about appointments, rewards to the efforts of patients in following the prescribed regimen, and stimulus of the support of family and friends are effective interventions to long course treatments [14]. These interventions might represent hard work, but they are cost-effective [30,33]. They have been associated to enhancement in adherence to long course treatments in 50%; and among those, 44.5% had improvement of prognosis [33]. Training of health personnel, healthcare education, community involvement, and epidemiological surveillance were part of the Cuban project that significantly reduced first and recurrent attacks of RF, severity of RHD, and direct costs of managing the disease, as well as increased compliance with secondary prophylaxis [5].

Inadequate frequency to appointments was found in 10% in the total sample of patients. This rate was 16% in a study in a rural community in north of India [19]. The inadequate frequency to appointments was 8% in the group who was successfully followed-up and 14.5% in the group that was lost to follow-up. There is a significant difference (p = 0.022) between the two groups, which might indicate irregularity in appointments as an early sign of the possibility of loss of follow-up.

Inadequate frequency to appointments is correlated to lower indices of adherence to treatment prescribed and it is the first sign of possible discontinuation of treatment, which is the most severe form of lack of adherence [17].

One hundred eighty patients (33.5%) were lost to follow-up. In another study in Rio de Janeiro, Brazil, at Hospital Universitário Antônio Pedro, 55% of patients were lost to follow-up in a group treated with parenteral secondary prophylaxis and 10% in another group treated with oral secondary prophylaxis [34].

Patients who were lost to follow-up in our center were not tracked because of the lack of a recall system. However, a rate of loss of follow-up of one third is significant and justifies the implementation of such a system. It would have a relatively low cost for the health service but a high possible repercussion in patients' morbidity and mortality.

This study has some limitations, as the impossibility to establish the causes of non-adherence to secondary prophylaxis, since it was retrospective; as well as the lack of further follow-up in one third of the patients, making it impossible to determine a more precise rate of recurrences.

The strengths of this study are the number of patients studied, one of the biggest cohorts published in RF; the determination of adherence rates to RF secondary prophylaxis in a representative Brazilian cohort; and the evidence supporting the necessity of a revision of Jones criteria for areas that still have high incidences of RF, as pointed out by several other authors.

## Conclusion

Based in the high rates of non-adherence to appointments and secondary prophylaxis, which lead to recurrent episodes of RF, we recommend implementation of a registry, and a system of active search of missing patients in every service responsible for the follow-up of RF patients. Measures to increase adherence to secondary prophylaxis involving patients and families need to be implemented formally, once non-adherence to secondary prophylaxis is the main cause of RF recurrence, still an issue in many parts of the world. Detection of irregularity in secondary prophylaxis or in appointments should be an alert about the possibility of loss of follow-up and closer observation should be instituted. Caution in strictly following Jones criteria to diagnose RF in countries with high incidence of the disease, as in Brazil, is recommended.

## Competing interests

The authors declare that they have no competing interests.

## Authors' contributions

CFP and JMT carried out the review of patients charts and drafted the manuscript. CFP conceived of the study, and participated in its design and coordination. SKFO and JMT participated in the design of the study. CFP and JMLB performed the statistical analysis. CFP, SKFO, and JMLB were involved in the writing and reviews of the final manuscript. All authors read and approved the final manuscript.
